# Perforators in the Leg: Why It Is Important for Orthopaedic Surgeons to Know

**DOI:** 10.5704/MOJ.1703.014

**Published:** 2017-03

**Authors:** N Mohd-Yusof, AA Ahmad-Alwi

**Affiliations:** Department of Orthopaedics, International Islamic University Malaysia Kulliyyah of Medicine, Kuantan, Malaysia

Dear Editor,

Orthopaedic surgeons have in-depth knowledge of the anatomy and blood supply of the bone. However, their knowledge of blood supply of the skin is minimal as compared to our plastic surgery colleagues. Knowing the blood supply of the skin can help orthopaedic surgeons minimize wound complications and facilitate plastic surgeons in managing open fracture wounds when such are referred.

Skin blood supply and the angiosome concept

Blood supply to the skin originates from the main artery artery of the leg. They give out branches that pass through the muscle or septum before perforating the deep fascia to supply an area of skin. This small territory of skin that is supplied by a perforator vessel is called an angiosome. The perforators are quite constant in their location and have been mapped out by many researchers. Perforators flaps are designed based on these perforating vessels as pivot points. By using a hand-held Doppler ultrasound, surgeons are able to identify the approximate location of the perforators, and reliably raise a flap. Identifying the location of the perforators is therefore important for orthopaedic surgeons to avoid damage during wound extension or undermining for skin closure.

**Fig. 1:**
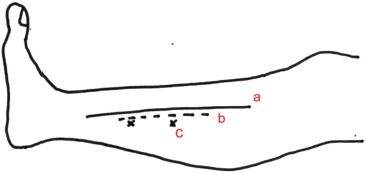
Schematic drawing of the leg showing (a) medial border of tibia, (b) recommended line for wound extension which is about one centimetre from the medial border of tibia and (c) the approximate location of the perforators which is about 10 centimetres and 15 centimetres from the medial malleolus.

**Fig. 2:**
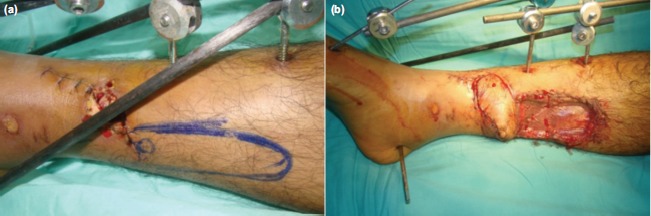
(a) Clinical photograph of a patient with grade IIIA open fracture of distal tibia. The wound extension goes beyond the one centimetre of the medial tibial border. Fortunately, the incision missed the perforators. The marking shows the location of the perforators. (b) Clinical photograph showing the wound after it was covered by the distal-based perforator flap. The donor site was skin grafted.

**Fig. 3:**
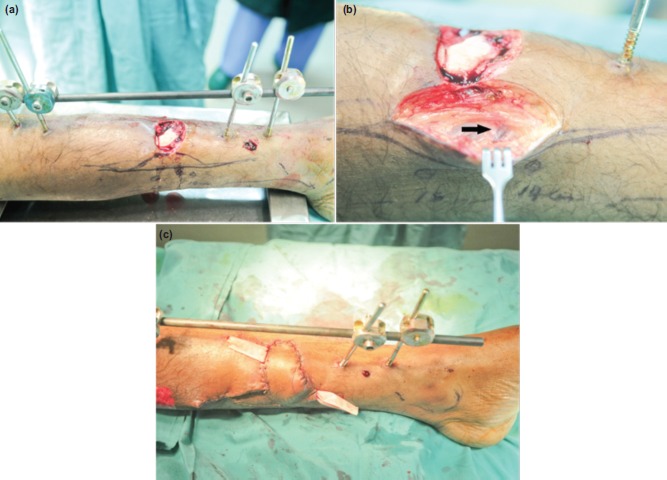
(a) Clinical photograph of a second patient with grade IIIB open fracture of the left distal tibia. Circles indicating identified PTA perforators using a handheld Doppler. (b) Perforator vessels (black arrow) can be seen entering the skin inferiorly through the exploratory incision. (c) Flap inset and split skin graft closure of the donor site. Two Penrose drains are placed to drain potential haematoma.

One of the common perforators that has been widely used is that arising from the posterior tibial artery (PTA). It is located about 10 cm proximal to the medial malleolus, halfway between the medial border of tibia and Achilles tendon (Fig. 1). Perforator flaps based on these vessels can be used to cover wound defect at the distal third of tibia (Fig. 2a, b and Fig. 3a, b, c). They have lower complications than the distally- based sural flap and avoid sacrifice of a major cutaneous nerve.

We would like to recommended that in the management of open fractures, wound extension or undermining should not go beyond one centimeter from the medial border of tibia to preserve the PTA perforators (Fig. 2).
